# At the Gate of Mutualism: Identification of Genomic Traits Predisposing to Insect-Bacterial Symbiosis in Pathogenic Strains of the Aphid Symbiont *Serratia symbiotica*

**DOI:** 10.3389/fcimb.2021.660007

**Published:** 2021-06-29

**Authors:** François Renoz, Vincent Foray, Jérôme Ambroise, Patrice Baa-Puyoulet, Bertrand Bearzatto, Gipsi Lima Mendez, Alina S. Grigorescu, Jacques Mahillon, Patrick Mardulyn, Jean-Luc Gala, Federica Calevro, Thierry Hance

**Affiliations:** ^1^ Biodiversity Research Centre, Earth and Life Institute, Université catholique de Louvain (UCLouvain), Louvain-la-Neuve, Belgium; ^2^ Institut de Recherche sur la Biologie de l’insecte, UMR 7261, CNRS, Université de Tours, Tours, France; ^3^ Center for Applied Molecular Technologies, Institute of Experimental and Clinical Research, Université catholique de Louvain (UCLouvain), Woluwe-Saint-Lambert, Belgium; ^4^ Univ Lyon, INSA-Lyon, INRAE, BF2i, UMR203, F-69621, Villeurbanne, France; ^5^ Louvain Institute of Biomolecular Science and Technology (LIBST), Université catholique de Louvain (UCLouvain), Louvain-la-Neuve, Belgium; ^6^ Walloon Center of Industrial Biology, Université de Liège, Liège, Belgium; ^7^ Laboratory of Food and Environmental Microbiology, Earth and Life Institute, Université catholique de Louvain (UCLouvain), Louvain-la-Neuve, Belgium; ^8^ Evolutionary Biology and Ecology, Université Libre de Bruxelles, Brussels, Belgium

**Keywords:** aphid symbiont, bacterial mutualism, genome evolution, metabolic pathways, pathogen, secretion systems, *Serratia symbiotica*, virulence factors

## Abstract

Mutualistic associations between insects and heritable bacterial symbionts are ubiquitous in nature. The aphid symbiont *Serratia symbiotica* is a valuable candidate for studying the evolution of bacterial symbiosis in insects because it includes a wide diversity of strains that reflect the diverse relationships in which bacteria can be engaged with insects, from pathogenic interactions to obligate intracellular mutualism. The recent discovery of culturable strains, which are hypothesized to resemble the ancestors of intracellular strains, provide an opportunity to study the mechanisms underlying bacterial symbiosis in its early stages. In this study, we analyzed the genomes of three of these culturable strains that are pathogenic to aphid hosts, and performed comparative genomic analyses including mutualistic host-dependent strains. All three genomes are larger than those of the host-restricted *S. symbiotica* strains described so far, and show significant enrichment in pseudogenes and mobile elements, suggesting that these three pathogenic strains are in the early stages of the adaptation to their host. Compared to their intracellular mutualistic relatives, the three strains harbor a greater diversity of genes coding for virulence factors and metabolic pathways, suggesting that they are likely adapted to infect new hosts and are a potential source of metabolic innovation for insects. The presence in their genomes of secondary metabolism gene clusters associated with the production of antimicrobial compounds and phytotoxins supports the hypothesis that *S. symbiotia* symbionts evolved from plant-associated strains and that plants may serve as intermediate hosts. Mutualistic associations between insects and bacteria are the result of independent transitions to endosymbiosis initiated by the acquisition of environmental progenitors. In this context, the genomes of free-living *S. symbiotica* strains provide a rare opportunity to study the inventory of genes held by bacterial associates of insects that are at the gateway to a host-dependent lifestyle.

## Introduction

Many insects thriving on unbalanced diets have evolved intimate relationships with symbiotic bacteria that are maternally inherited throughout generations and can influence many aspects of their hosts’ biology (Engel and Moran, 2013; McFall-Ngai et al., 2013; Oliver et al., 2014). Certain symbionts are obligate nutritional partners for their host because they produce essential nutrients that are deficient in their diet, such as amino acids and vitamins (Douglas, 1998; Russell et al., 2017). Insects can also harbor facultative bacterial partners with various effects on the host phenotype, depending on the ecological context (Oliver et al., 2010; Lo et al., 2016). It is now well established that those symbionts, whether obligate or facultative, evolved from originally free-living bacterial lineages (Husník et al., 2011; Clayton et al., 2012; Manzano-Marín et al., 2020) and that adaptation to a host-restricted lifestyle is accompanied by a reductive evolution of their genome (van Ham et al., 2003; Burke and Moran, 2011; Nikoh et al., 2011; Sloan and Moran, 2012; Latorre and Manzano-Marín, 2017). The isolation of bacterial strains capable of living freely outside of insects but related to insect endosymbionts (including members of the genera *Sodalis* and *Arsenophonus*) suggests the presence of insect symbiont progenitors in the environment (Bressan et al., 2009; Clayton et al., 2012). In addition to this, recent experimental work on plant-sucking stinkbugs (Insecta: Hemiptera) has demonstrated that extracellular gut symbionts of mutualistic nature residing in specialized gut-associated structures arise from free-living bacteria taken up from the environment (Kikuchi et al., 2011; Hosokawa et al., 2016a; Hosokawa et al., 2016b). These findings provide tangible evidence for the existence of an environmental pool of bacteria from which new intimate partnerships between insects and bacteria can emerge and identifying potential progenitors of endosymbionts is essential to gain finer insight into the multiple evolutionary trajectories leading to bacterial mutualism.

Aphids (Hemiptera: Aphididae) have long been model systems for addressing functional diversity and evolution of bacterial mutualism in insects (Buchner, 1965; Feldhaar, 2011; Baumann et al., 2013). Almost all aphids harbor an obligate symbiont, *Buchnera aphidicola*, that provides essential amino acids and vitamins lacking in the plant phloem sap (Douglas, 1998; Russell et al., 2017). *B. aphidicola* symbionts have a highly reduced and static genome, resulting from about 150 millions of years of coevolution (van Ham et al., 2003) and isolation in dedicated insect cells called bacteriocytes (Braendle et al., 2003). In some aphid species, *B. aphidicola* has lost some metabolic capabilities and nutrition also relies on co-obligate partners (Manzano-Marín et al., 2016; Meseguer et al., 2017; Monnin et al., 2020). In addition to nutritional symbionts, many aphid species can be associated with heritable facultative symbionts that can have various effects on the host phenotype, such as alteration of the body color (Tsuchida et al., 2010), tolerance to heat-stress (Burke et al., 2010) or protection from natural enemies (Scarborough et al., 2005; Oliver et al., 2009). These facultative associations are often regarded as intermediate stages between the free-living and the mutualistic lifestyle (Latorre and Manzano-Marín, 2017). Indeed, compared to *B. aphidicola*, facultative symbionts hold moderately reduced genomes and often retain mechanisms similar to those of opportunistic pathogens to infect a wider variety of host tissues and colonize new hosts (Dale and Moran, 2006; Degnan et al., 2009).

The aphid-associated symbiont *Serratia symbiotica* has become a valuable model for dissecting the mechanisms underlying endosymbiosis emergence and evolution because the diversity of *S. symbiotica* reported to date provides a comprehensive picture of the various associations that bacteria can share with insects. Reported *S. symbiotica* strains are associated with various host phenotypes and hold genomes of contrasting sizes and features reflecting their lifestyle, which ranges from the free-living lifestyle to the obligate intracellular mutualism (Burke and Moran, 2011; Foray et al., 2014; Manzano-Marín and Latorre, 2016; Latorre and Manzano-Marín, 2017). For instance, strains of *S. symbiotica* associated with the pea aphid *Acyrthosiphon pisum* (subfamily Aphidinae) hold mildly-reduced genomes and are of a facultative nature as they provide protective effects against high temperatures and parasitoid wasps (Oliver et al., 2003; Burke et al., 2010; Burke and Moran, 2011) and may have some nutritional benefits (Koga et al., 2003; Nikoh et al., 2019). Other strains of *S. symbiotica* associated with aphid species of the subfamilies Lachninae and Chaitophorinae are involved in co-obligate associations, hold rather small genomes and compensate for the reduction of *B. aphidicola*’s metabolic capacity for the biosynthesis of nutrients, particularly biotin (vitamin B_7_), riboflavin (vitamin B_2_) and the amino acid tryptophan (Lamelas et al., 2011a; Lamelas et al., 2011b; Manzano-Marín and Latorre, 2014; Manzano-Marín and Latorre, 2016; Manzano-Marín et al., 2016; Manzano-Marín et al., 2017; Meseguer et al., 2017; Manzano-Marín et al., 2018; Monnin et al., 2020). Genomic analyses have indicated that this dependence of aphids on co-obligate strains has likely appeared independently on many occasions in the evolutionary history of aphids and has evolved in a very dynamic fashion, with *S. symbiotica* being acquired and replaced several times during the diversification of their host aphids (Manzano-Marín et al., 2017; Meseguer et al., 2017; Manzano-Marín et al., 2020). The recruitment of new bacterial partners and the repeated replacement of pre-existing intracellular symbionts is now considered as a recurrent evolutionary phenomenon that occurs in many phloem-feeding insects (Koga and Moran, 2014; Husnik and McCutcheon, 2016; Mao and Bennett, 2020).

Although there is compelling evidence that new mutualistic associations between insects and bacteria are continually forming in nature (Kikuchi et al., 2011; Clayton et al., 2012; Hosokawa et al., 2016a; Hosokawa et al., 2016b; Matsuura et al., 2018), little is known about how this occurs in aphids. This is partly due to the fact that a very limited number of free-living bacteria displaying features allowing identifying them as putative ancestors of facultative and obligate intracellular symbionts have been discovered so far. However, several *S. symbiotica* strains with free-living abilities have recently been discovered and they offer the rare opportunity to study the inventory of genes held by insect-associated bacteria just prior to the acquisition of an host-restricted lifestyle (Foray et al., 2014; Grigorescu et al., 2018; Renoz et al., 2020). Compared to their intracellular facultative and co-obligate relatives that are sheltered in bacteriocytes and/or sheath cells, these culturable *S. symbiotica* strains harbor larger genomes (Foray et al., 2014; Renoz et al., 2020) and have the propensity to rapidly invade the digestive tract of aphids (Renoz et al., 2015; Pons et al., 2019b). Once they have invaded their host’s gut, these strains cause fitness costs and become true gut pathogens, unable to establish persistent maternal transmission and transmitted primarily *via* the fecal-oral route (Pons et al., 2019b; Perreau et al., 2021). These strains are able to invade plants and circulate through the phloem sap before infecting aphids (Pons et al., 2019a). Interestingly, in a complementary fashion, field studies have shown that *S. symbiotica* can naturally reside in plants as well as in the digestive tract of various insect groups (Renoz et al., 2019; Pons et al., 2021, pre-print). All these findings suggest the existence of *S. symbiotica* strains that have the ability to circulate in different environmental compartments and to be acquired horizontally, and are therefore potential progenitors of stably maintained mutualistic endosymbionts. To shed light on the origin of the aphid symbiont *S. symbiotica* and the genomic traits that could predispose to the acquisition of a host-restricted lifestyle (i.e., exaptations in *S. symbiotica* for aphid symbiosis), we improved and analyzed the whole-genome sequences of three *S. symbiotica* strains that retain free-living abilities and isolated from different aphid strains and species, and performed comparative genomic analyses that also include the genomes of 12 host-restricted *S. symbiotica* strains. Our study suggests that the mutualistic strains associated with aphids have evolved from *S. symbiotica* strains that originally inhabited plants. The three culturable strains still hold a wide range a virulence factor that may explain their pathogenicity to aphids, but are at the same time characterized by genomic traits that predispose them to become mutualistic partners.

## Materials and Methods

### Genome Sequencing and Assemblies

The whole-genome sequences of three *S. symbiotica* strains previously published (Foray et al., 2014; Renoz et al., 2020) were improved as described below. The whole-genome sequence of the *S. symbiotica* strain CWBI-2.3^T^ (hereafter SsAf2.3) isolated from the black bean aphid *Aphis fabae* (Foray et al., 2014) was improved using the Pacific Biosciences RS sequencing technology (KeyGene, Wageningen, Netherlands). A 20-kb library was sequenced on two single-molecule real-time (SMRT) cells. The reads were assembled using HGAP 2.1 *de novo* assembly pipeline. The few final gaps and long repeats found in the resulting draft genome were then covered by PCR. To validate the presence and size of the plasmids identified *in silico*, a plasmid profile was determined using a modified Eckhardt agarose gel electrophoresis approach (Eckhardt, 1978).

The genomes of the *S. symbiotica* strains 24.1 (hereafter SsAf24.1) isolated from *A. fabae* and Apa8A1 (hereafter SsApa8A1) isolated from the sage aphid *Aphis passeriniana* (Grigorescu et al., 2018) had been previously sequenced by whole-genome paired-end sequencing using Illumina on a HiSeq 4000 platform with 2 × 100 paired-end reads (Illumina, San Diego, CA, USA) and a sequencing depth >100x (Renoz et al., 2020). These data were complemented by long reads obtained using a ONT MinION sequencer. Prior to the MinION nanopore sequencing, the integrity of genomic DNA was evaluated using an Agilent 2200 TapeStation with the Genomic DNA ScreenTape (Agilent). Both samples displayed a DNA Integrity Number value ≥9.5. Libraries were then prepared from 400 ng of genomic DNA using the Rapid Barcoding Sequencing kit (SQK-RBK004) protocol (Oxford Nanopore Technologies). Briefly, the DNA molecules were cleaved by a transposase and barcoded tags were attached to the cleaved ends. The barcoded samples were then pooled and Rapid Sequencing Adapters were added to the tagged ends. The pooled libraries were sequenced into a FLO-MIN106 (R9.4.1) flow cell for a 48h run according to manufacturer’s instruction. After the run, fast5 files were basecalled on the MinIT using default settings in MinKNOWv18.12 and Guppy v3.0.3 and a High Accuracy (HAC) Flip-Flop methodology. Finally, a hybrid assembly strategy combining the data generated by the Illumina and ONT MinION sequencing approaches was performed using Unicycler v0.4.8 (Wick et al., 2017). Raw sequence reads have been deposited in the European Nucleotide Archive (studies with accession numbers: PRJEB44297 for SsAf2.3, PRJEB44257 and PRJEB44266 for SsAf24.1, and PRJEB44268 and PRJEB44273 for SsApa8A1).

### Genome Annotation and Comparative Analyses

**Figure 1:**
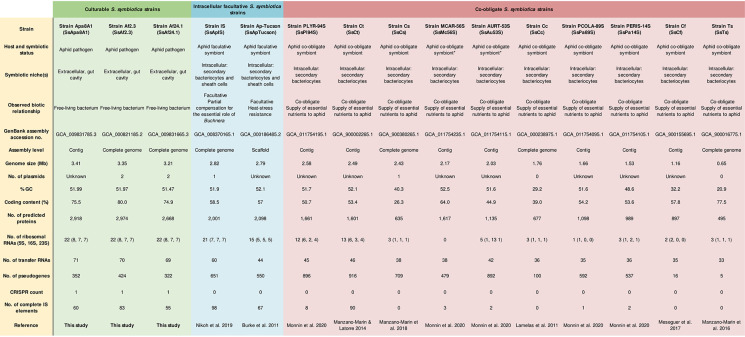
General genomic features of *S. symbiotica* strains ordered by genome size. *The nature of the relationship between aphids and strains SsMc56S and SsAu53S is currently under debate.

The three genomes were annotated with the Prokaryotic Genome Annotation Pipeline (PGAP) (Tatusova et al., 2016). To perform genomic comparative analyses that include the other available sequenced genomes of *S. symbiotica*, the datasets corresponding to their annotation were downloaded from NCBI (GenBank annotations were preferred when available) (**Figure 1**). The pan- and core-genomes were computed using the EDGAR software platform (Blom et al., 2016) and the protein sequences were functionally annotated using eggNOG mapper v2 (Huerta-Cepas et al., 2017). The fifteen available genomes of *S. symbiotica* were deposited on the MicroScope platform (Médigue et al., 2019; Vallenet et al., 2020) and their metabolic capabilities compared using MicroCyc, a collection of microbial Pathway/Genome Databases (PGDBs) based on the MetaCyc database and specifically dedicated to the analysis of microbial pathways (Caspi et al., 2020). Metabolic reconstructions generated by MicroCyc were manually verified and CDSs were considered as pseudogenes when their length was less than 80% of the one of their orthologues in the other genomes (Lerat and Ochman, 2005; Burke and Moran, 2011). AntiSMASH v5.0.0 and the MIBiG database were used to predict regions coding for complete secondary metabolites in the different genomes (Blin et al., 2019). Virulence genes were identified using the virulence factor database (VFDB, http://www.mgc.ac.cn/VFs/) (Chen et al., 2005; Liu et al., 2019): datasets of virulence genes were downloaded and BLASTp were performed (minimum 50% aa identity, 80% align. coverage). Complete secretion systems were identified with MacSyFinder (Abby et al., 2014). Insertion sequences (hereafter IS) predictions were generated with ISsaga (Varani et al., 2011). CRISPR-Cas systems were detected using CRISPRCasFinder and MacSyFinder (Abby et al., 2014; Couvin et al., 2018). Intact prophage regions were predicted with PHASTER (Arndt et al., 2016). Assemblies and annotations are available on Genbank under the following accession numbers: CP050857-CP050857 for SsAf2.3, CP072650-CP072652 for SsAf24.1 and WSPO00000000.3 for SsApa8A1.

### Molecular Phylogenetic Analyses

To infer the origin of the culturable *S. symbiotica* strains and to reconstruct the phylogenetic history of the symbiosis between aphids and *S. symbiotica*, we built a phylogenetic tree using a set of single-copy core concatenated protein sequences shared across all *S. symbiotica* strains and several *Serratia* spp. that are representative of the genus and include plant- and animal-associates (**Table S1**). All protein sequences of the different species and strains were analyzed with OrthoFinder (version 2.3.12) (Emms and Kelly, 2015; Emms and Kelly, 2019) to identify orthologues and orthogroups. A set of 221 single-copy genes present in each bacterial strain was then used for phylogeny estimation. The alignments of this set of genes were concatenated using FASconCAT-G (Kück and Meusemann, 2010). By initially defining each gene as a separate partition, we performed a ModelFinder analysis (Kalyaanamoorthy et al., 2017) on the concatenated alignment with IQ-TREE (version 1.6.12) (Nguyen et al., 2015); (options -m TESTMERGEONLY and -rcluster 10; set of models evaluated: mrbayes) to select the best partitioning of the alignment and the best model for each partition (-p option, allowing each partition to have its own evolution rate). A maximal likelihood analysis was then conducted on the best partitioning and modelling scheme (-p option) with IQ-TREE (MPI version). Branch support was assessed using ultrafast bootstrapping (UFBoot; (Minh et al., 2013; Hoang et al., 2018) and an approximate likelihood ratio test (SH-aLRT; (Anisimova et al., 2011).

## Results and Discussion

### Genome Sequencing of the Culturable *S. symbiotica* Strains

The sequencing of the SsAf2.3 strain using the Pacific Biosciences RS sequencing technology yielded 144,745 reads for a mean read length of 6,126 bp and resulted in a 210x average genome coverage. This allowed us to obtain an improved genome assembly version compared to the one previously published (Foray et al., 2014) in that the new version offered us the opportunity to circularize the chromosome and to identify two plasmids, namely pSsAf2.3-1 (82.6 kb, 57 CDSs) and pSsAf2.3-2 (116.7 kb, 121 CDSs). The presence and size of the plasmids identified *in silico* were validated by a plasmid profile generated by a modified Eckhardt agarose gel electrophoresis approach (Eckhardt, 1978) (**Figure S1**). The sequencing of the strains SsAf24.1 and SsApa8A1, performed using the MinION nanopore sequencing approach, generated a total of 944,030 and 686,673 reads with an average read length of 4.04 and 8.18 kb, respectively. Combining Illumina and MinION sequencing data using Unicycler improved the previously published genomes. This allowed us to circularize the chromosome of the SsAf24.1 strain and to identify to plasmids, namely pSsAf24.1-1 (104.9 kb, 106 CDSs) and pSsAf24.1-2 (54.8 kb, 47 CDSs). The SsApa8A1 genome consists of 7 contigs. Both genomes were associated with a high coverage with Illumina (>100x) and MinION data (>1000x).

### General Genomic Features of Culturable *S. symbiotica* Strains

The culturable strains SsAf2.3, SsAf24.1 and SsApa8A1 exhibit a similar genome organization (size, %GC, coding content, number of tRNAs, etc.) (**Figure 1**). Their genomes have lengths comprised between 3.2 and 3.4 Mb, and contain between 2,500 and 3,000 CDSs covering about 75% of the entire genome. They are larger than those of the intracellular facultative and co-obligate *S. symbiotica* strains sequenced so far and contain a higher proportion of coding DNA. However, compared to genome sizes of other *Serratia* species, which range from 4.9 to 5.6 Mb, the ones of the three culturable strains analyzed in our work are remarkably smaller (**Table S1**). Compared to the genomes of the opportunistic free-living pathogen *S. marcescens* Db11 (hereafter Db11) and intracellular co-obligate *S. symbiotica* strains, the genomes of the culturable *S. symbiotica* strains show significant enrichment in pseudogenes and IS elements (**Figure 1**), suggesting, despite their free-living capabilities, an ongoing reduction of their genome and a shift from a free-living to a host-restricted lifestyle (Moran and Plague, 2004; Toft and Andersson, 2010; Latorre and Manzano-Marín, 2017). Synteny’s analyses highlight an increase of rearrangements in that the genomes of culturable *S. symbiotica* strains compared to free-living close relatives of the genus (**Figure S2**).

**Figure 2:**
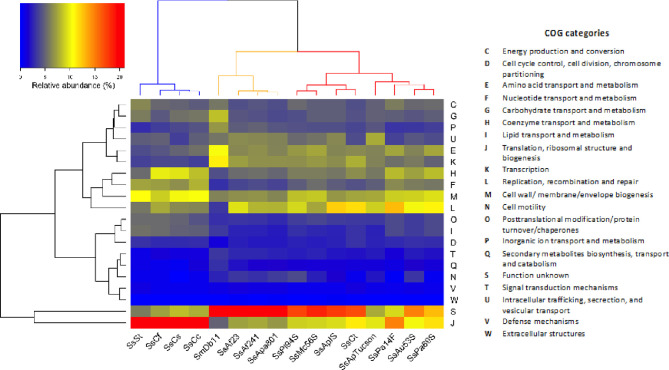
Functional profiles of selected *S. symbiotica* strains and *S. marcescens* Db11. Two-way hierarchical clustering heatmap showing the relative abundance of CDSs in each COG categories according to the total number of CDSs in each genome. Cladogram at the top highlights three main clusters among *S. symbiotica* strains: one cluster including only *S. symbiotica* from Clade B (left cluster); one cluster grouping all culturable strains with *S. marcescens* Db11 (middle cluster); and one cluster including facultative and co-obligate *S. symbiotica* strains (right cluster).

Among the CDSs that could be classified in at least one EGGNOG group, 95% (SsAf2.3), 96% (SsAf24.1) and 94% (SsApa8A1) were assigned to a biological function. The hierarchical clustering of COG (Clusters of Orthologous Groups of proteins) profiles reveals three main clusters among *S. symbiotic*a strains (**Figure 2**). Culturable strains compose one cluster with SmDb11, characterized by an enrichment of CDSs of unknown function (category S) that are probably involved in secondary and non-essential functions. The relative abundance of CDSs associated with virulence and motility (categories U and N, respectively) is also higher in the culturable strains than in the other intracellular host-restricted strains. A second cluster is composed of co-obligate *S. symbiotica* strains of clade B. As previously proposed (Manzano-Marín et al., 2012), the strains from this clade are featured by a streamlined genome conserving genes involved in basic cellular functions, like protein synthesis (category J). Category M is also strongly confirmed in this second cluster, as these strains conserved a capacity to synthetize their own cell membrane despite massive erosion of their genome. Finally, facultative and co-obligate strains compose a third cluster. They show intermediate profiles compared to the other two clusters, with moderate enrichment of housekeeping genes (category J) but still a large proportion of non-essential CDSs with unknown functions. Category L is also highly conserved in this cluster, as these strains retain CDSs involved in genome replication and repair systems. These results support the hypothesis that symbiotic bacteria clustered by GOC categories tend to have similar ecological niches (Santos-Garcia et al., 2014). However, phylogenetic history seems to play an important role as the co-obligate *S. symbiotica* strains of clade B constitute a separate cluster from the other co-obligate strains. On the other hand, it is also possible that the monophyly of these strains is the result of a long-branch attraction (LBA) artefact (Bergsten, 2005).

**Figure 3:**
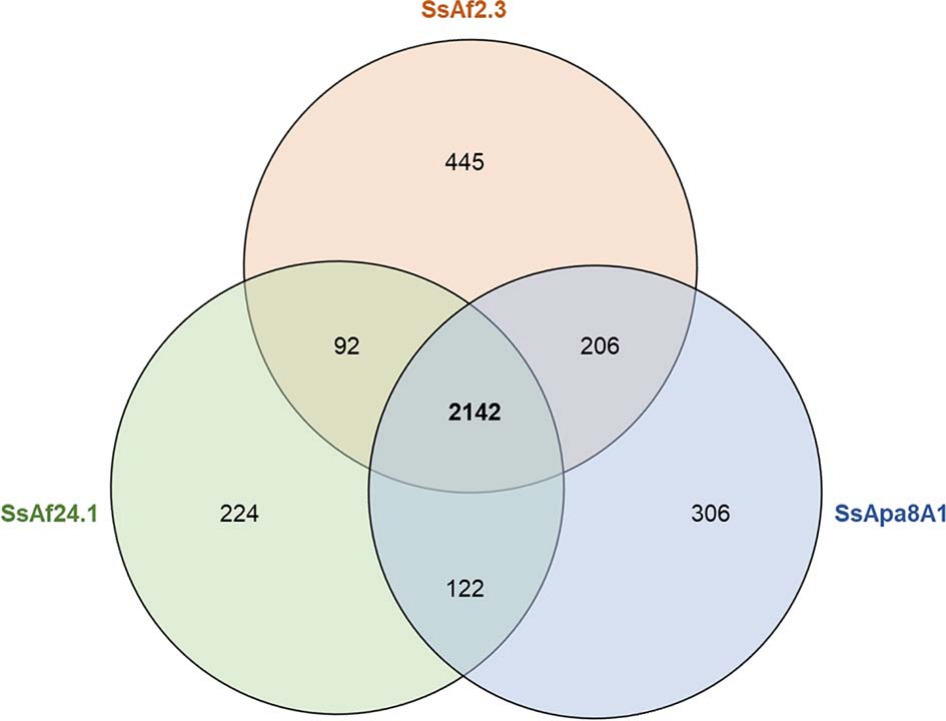
Venn diagram comparing the gene inventories of the three culturable *S. symbiotica* genomes computed by EDGAR (Blom et al., 2016) based on reciprocal best BLAST hits of the coding sequences predicted by PGAP (Tatusova et al., 2016).

The genomes of the three culturable strains share most genes and pathways with a core-genome composed of 2,142 CDSs (**Figure 3** and **Table S2**). The strain-specific genome of each strain constitutes only about 10% of the CDSs and most strain-specific genes encode CDSs with no assigned function (**Table S3**). The most notable differences between the strains were found in phage proteins, transposases, conjugal transfer proteins and in the proteins involved in the toxin–antitoxin (TA) systems (known to participate in plasmid stability), stress management, biofilm formation and antibiotic resistance (Van Melderen and Saavedra De Bast, 2009). Interestingly, the three genomes also differ in their respective repertoires of genes encoding proteins related to the flagellar apparatus. Indeed, the genome of SsApa8A1 contains many more flagellate-related genes than the genomes of SsAf2.3 and SsAf24.1. This point is discussed in more detail in the section devoted to the virulence factors encoded by the different *S. symbiotica* genomes. Overall, the genomes show few differences in the content of CDSs and little variation in pathways of considerable biological relevance, suggesting a single evolutionary origin of the three strains and a possible adaptation to similar niches: the digestive tract of the aphids and probably the phloem sap through which they can transit (Pons et al., 2019a; Pons et al., 2019b).

### Genomic Comparison Between Culturable and Intracellular Host-Restricted Strains

The core-genome defined from the fifteen *S. symbiotica* sequenced genomes is composed of 302 protein-coding genes, identified as housekeeping genes (**Table S4**). Beyond these genes, our analyses predicted that the *cvpA* (colicin V production accessory protein) gene is present in all the genomes (except the SsMC-56S genome for which a chromosome fragment is missing) (**Figure S3** and **Table S5**). *cvpA* encodes for an inner-membrane protein primarily described as a key factor for colicin V production by members of the Enterobacteriaceae to kill competing bacteria (Fath et al., 1989; Gérard et al., 2005; Zaini et al., 2008). However, the synthesis of colicin V also requires the expression of several plasmid-borne genes (*cvaA*, *cvaB*, *cvaC*, and *cvi*) (Gérard et al., 2005) that have not been detected in the genomes we studied here. An alternative role of *cvpA* in the context of bacterial mutualism has never been investigated, whereas *cvpA* has been conserved in the genomes of many endosymbiotic bacteria, including the obligate aphid symbiont *B. aphidicola* (Charles et al., 2011) and the obligate tsetse fly symbiont *Wigglesworthia* (Prickett et al., 2006), but also symbiotic bacteria associated with deep-sea tubeworms and corals (Gardebrecht et al., 2012; Carlos et al., 2014).

Recent studies have shown that *cvpA* contribute to host colonization by pathogenic bacteria. The *cvpA*-*purF* locus is required for intracellular replication of uropathogenic *E. coli* by promoting the biosynthesis of purine nucleotides necessary for survival in an intracellular niche (Shaffer et al., 2017). However, unlike *cvpA, purF* was lost in *S. symbiotica* strains with the most eroded genomes (**Figure S3** and **Table S5**), while it is essential for this biosynthesis of purine nucleotides. *cvpA* may also play a role in biofilm development (Hadjifrangiskou et al., 2012) and is required for colonization of the human intestine by enteric pathogens including *Vibrio parahaemolyticus* and *Escherichia coli* (Hubbard et al., 2016; Warr et al., 2021). It has been proposed that this membrane protein contributes to cell envelope homeostasis in response to hostile environmental conditions set up by the host to counter infection (Warr et al., 2021). In light of these recent findings, the conservation of *cvpA* in symbiotic bacteria, including those with an extremely small genome, may indicate that this gene helps bacterial symbionts cope with the different niches they may face, namely the gut, the hemolymph and the intracellular environment.

### Origin of Aphid-*S. symbiotica* Symbioses and Acquisition Routes

**Figure 4:**
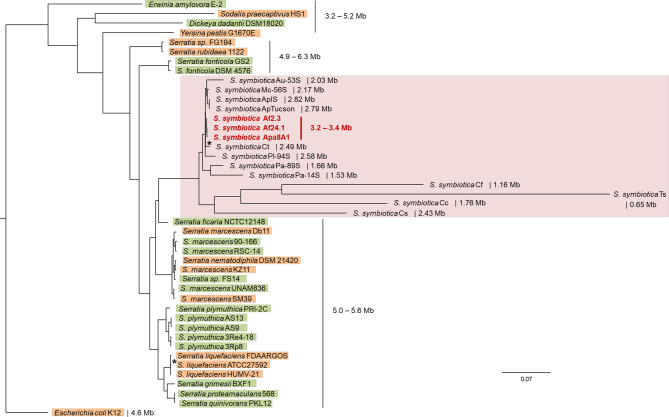
Phylogenetic positioning of the culturable *S. symbiotica* strains in the species and in the genus. The tree shown is the maximum likelihood topology inferred with IQ-TREE on 221 concatenated single-copy genes shared by all selected strains. Branch support (SH-aLRT and ultrafast bootstrap values) was > 99% for all nodes, except for two nodes associated with support values > 80% (*): (*S. symbiotica* Af2.3, *S. symbiotica* Af24.1, *S. symbiotica* Apa8A1 and *S. symbiotica* Ct) and *S. liquefaciens* FDAARGOS and *S. liquefaciens* ATCC27592). The *S. symbiotica* strains are highlighted in pink and the three cultured strains are denoted in bold red. *Serratia* members found associated with plants are highlighted in green and *Serratia* members found associated with animals are highlighted in orange. Partitions and associated models in a nexus file available in **Supplementary Material**.

There is increasing evidence to support the hypothesis that host-restricted symbionts are derived from free-living progenitors acquired in the environment and having been gradually and obligatorily integrated into the insect’s physiology and development (Kikuchi et al., 2007; Moya et al., 2008; Clayton et al., 2012; Hosokawa et al., 2016a; Manzano-Marín et al., 2020). The genus *Serratia* includes members living in a wide range of habitats including water, soil, plants, humans and invertebrates (Petersen and Tisa, 2013). Here, we first examined the evolutionary relationship between the three culturable strains with the other members of the genus and assessed the origin of relationships involving *S. symbiotica* by estimating a phylogenetic tree. This was based on the use of a set of single-copy core concatenated protein sequences shared between 1) all *S. symbiotica* strains, 2) strains representative of the diversity of the genus and reported as being associated with plants or animals, and 3) several other members of the Enterobacteriaceae. The fifteen *S. symbiotica* strains form a well-supported monophyletic group, sister to the groups of *Serratia* species described as plant and animal associates (**Figure 4**). However, the tree topology did not allow us to deduce the lifestyle of the last “common ancestor” of the *S. symbiotica* strains, i.e. whether this was associated with plants or animals, because the nature of the associated host seems to be versatile among the genus *Serratia* (**Figure 4**). Actually, *Serratia ficaria*, the closest relative of *S. symbiotica*, was originally found associated with fig trees, but the species remain poorly described and seems to be able to infect humans (Grimont et al., 1981; Anahory et al., 1998).

To infer the origin of *S. symbiotica*, we therefore used a second strategy and examined the presence of gene clusters involved in the biosynthesis of secondary metabolites in the different genomes of *S. symbiotica*. Indeed, these gene clusters are supposed to reflect the environment that bacteria face and therefore to which they are adapted (Ziemert et al., 2014). Our analyses showed that several *S. symbiotica* genomes harbor gene clusters predicted to be intact and involved in the biosynthesis of secondary metabolites, particularly the genomes of the three culturable strains (**Table S6**). These bacteria house a gene cluster involved in the biosynthesis of massetolide A, a cyclic lipopeptide with antifungal properties that has been reported to be produced by endophytic strains of *Pseudomonas* and to contribute to host plant invasion (Tran et al., 2007; de Bruijn et al., 2008; Raaijmakers et al., 2010; Loper et al., 2012). Interestingly, the genome of the co-obligate strain SsCt also harbors a gene cluster involved in the biosynthesis of this lipopeptide. Similarly, the genome of strain SsAf2.3 contains a gene cluster involved in the synthesis of syringopeptin, a phytotoxin produced by members of the genus *Pseudomonas* and contributing to plant invasion (Ballio et al., 1991; Weeraratne et al., 2020). The genome of SsAf24.1 harbors a cluster involved in the synthesis of rhizomides, which are compounds associated with antimicrobial and cytotoxic properties and are notably produced by members of the genera *Pseudomonas* and *Burkholderia* (Wang et al., 2018; Kunakom and Eustáquio, 2019; Niem et al., 2019). The SsAf2.3 and SsApa8A1 genomes both host a gene cluster involved in the synthesis of luminmide, a cyclic pentapeptide associated with cytotoxic effects on eukaryotic cells (Li et al., 2019).

**Figure 5:**
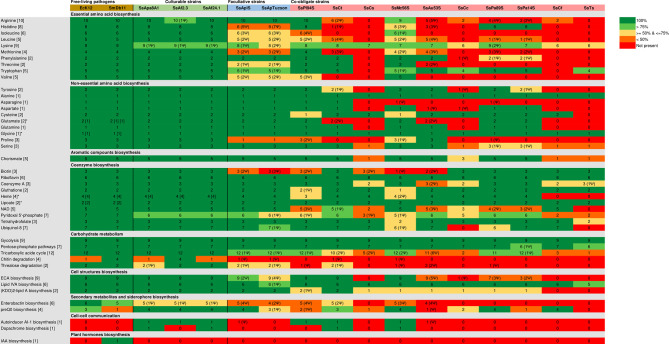
Comparison of metabolic genes repertories among the different *S. symbiotica* strains. The minimal number of genes for a metabolic pathway is shown in each of the brackets. Color represents the completeness of the MetaCyc pathways: dark green for 100%, light green for 99-75%, yellow for 74-50%, orange for 50-1% and red for 0%. Asterisk (*) denotes that the bacterium possesses a complete alternative pathway for the biosynthesis of the final product with the minimal number of genes for the alternative pathway indicated in the brackets. The numbers followed by Ψ in the round brackets indicate the number of pseudogenes found for the pathway.

The presence in *S. symbiotica* genomes of gene clusters involved in the biosynthesis of certain secondary metabolites such as massetolide A and syringopeptin suggest that certain *S. symbiotica* members could have interacted and/or are still interacting with plants and that *S. symbiotica* symbionts residing in aphids may have evolved from strains that originally inhabited plants. Along the same lines, it has recently been demonstrated that SsAf2.3 can infect the roots of *Vicia faba*, before moving to the phloem sap and subsequently infecting the aphids that feed on it (Pons et al., 2019a). In addition, *S. symbiotica* has been detected from tissues of plants collected in the field (Pons et al., 2021, pre-print). In the light of these observations, we propose that plants may serve as alternative hosts and vectors for the free-living *S. symbiotica* strains, some of which can become intestinal associates of aphids before evolving into mutualistic symbionts. This scenario is further supported by the great versatility of members of the genus *Serratia*, which can be associated with both animals and plants [e.g. *S. marcescens*, which includes strains pathogenic to insects and plants as well as strains considered beneficial endophytes of plants (Petersen and Tisa, 2013; Eckelmann et al., 2018)]. Yet, the exact nature of the interaction between certain *S. symbiotica* strains and plants remains to be further studied: although it is assumed that cultivable strains may be associated with positive effects on these hosts by stimulating their growth (Pons et al., 2019a), they are unable to synthesize indole-3-acetic acid (IAA) (**Figure 5**), a plant hormone produced by many endophytic *Serratia* members (Neupane et al., 2012; Khan et al., 2017). Ongoing studies should clarify the nature of the relationships that culturable *S. symbiotica* strains have with plants and the effects associated with the secondary metabolites they may produce. Furthermore, a hypothesis that cannot be excluded is that these secondary metabolites are toxic to insects and contribute to the virulence of certain *S. symbiotica* strains. In such a context, culturable *S. symbiotica* strains represent fascinating models for understanding how bacteria develop cross-kingdom host jumps and exploit multiple hosts.

### Metabolic Capacities of Culturable *S. symbiotica* Strains

Compared to the facultative and co-obligate strains, the three culturable *S. symbiotica* strains display larger metabolic capabilities, that include intact pathways and then the potential to produce amino acids and vitamins. Indeed, all culturable strains are potentially capable of biosynthesizing most amino acids considered as essentials for metazoan cells, with a few exceptions (**Figure 5**). The *argD* gene is pseudogenized in all three genomes. However, the N-acetylornithine aminotransferase activity in lysine synthesis can also be achieved by the product of *astC* (present in the genomes of SsAf24.1 and that of SsApa8A1) and *SerC* (Lal et al., 2014) present in the genome of all three culturable strains, suggesting that all three strains are likely still capable to synthetize arginine and lysine.

The genomes of all three culturable strains have also intact pathways for the biosynthesis of all non-essential amino acids, as well as for many coenzymes. However, the *epd* gene, which is required for the erythrose-4-phosphate dehydrogenase activity in the biosynthesis of pyridoxal 5’-phosphate (the active form of vitamin B_6_), is absent from their genomes whereas it is present in the entomopathogens *Escherichia coli* K12 (EcK12) and SmDb11. However, this dehydrogenase activity may be performed by the product of *gapA* (Zhao et al., 1995), that is present and intact in all *S. symbiotica* genomes.

The metabolic pathways exhibited by *S. symbiotica* reflect the evolutionary trajectories of the strains and their contribution to the respective symbiotic system they are involved in. For example, the co-obligate strains associated with aphid species of the subfamilies Lachninae (SsCt, SsCs, SsCc, SsCf and SsTs) and Chaitophorinae (SsPl-94S, SsPa-89S and SsPa-14S) retained the capacity of producing biotin (B_7_ vitamin) and thus compensate for *B. aphidicola*’s inability to synthesize that coenzyme (Manzano-Marín and Latorre, 2016; Manzano-Marín et al., 2016; Manzano-Marín et al., 2017; Manzano-Marín et al., 2020) while the facultative strains SsApIS and SsApTucson associated with *A. pisum* have lost this ability (**Figure 5**). The larger metabolic potential of culturable strains for the biosynthesis of amino acids, vitamins and other coenzymes supports the hypothesis that those undergo the early stages of host adaptation and could be a source of metabolic innovations in emerging mutualistic associations.

Finally, another notable aspect concerning the metabolic capacities of three culturable *S. symbiotica* strains concerns their capacity to degrade chitin. SsAf2.3 is the only strain potentially capable of degrading chitin (**Figure 5**), the main component of the peritrophic matrix (PM) of the midgut that provides protection for the underlying epithelium from abrasion, toxins and pathogens (Hegedus et al., 2009; Merzendorfer et al., 2016). Many entomopathogenic members of the genus *Serratia* are capable of perforating the PM of insects *via* chitinases (Regev et al., 1996). However, SsAf2.3 was negative in tests for chitinase (Sabri et al., 2011).

### Phages and CRISPR Arrays in the *S. symbiotica* Genomes

Genes that are important for host invasion and adaptation (e.g., genes encoding secretion systems and virulence factors) are often found in close proximity to mobile elements such as plasmids and phages that serve as intermediaries for the exchange of genetic material and the introduction of new functions into bacterial genomes (Clokie et al., 2011; Weldon and Oliver, 2016). In the context of the evolution of bacterial mutualism, bacteria in the early stages of host adaptation tend to harbor more phage regions than bacteria involved in the advanced stages of the process (Toft and Andersson, 2010). As expected, more intact phage regions were found in the genomes of the culturable strains than in the genomes of facultative and co-obligate strains (**Table S7**). In the symbiotic bacteria associated with aphids, increased attention has been paid to the lysogenic phage APSE (*Acyrthosiphon pisum* secondary endosymbiont), often found in the facultative symbiont *Hamiltonella defensa* where its presence is associated with protective effects against parasitoid wasps to the benefit of the aphid host (Oliver et al., 2009; Weldon and Oliver, 2016). This protection is based on bacteriophage-encoded toxins that hinder the development of parasitoid larvae (Oliver et al., 2012). No APSE-related regions were detected in the genomes of the three culturable strains as well as in the genomes of the intracellular strains, with the exception of SsCt where a region for which the most common phage hit was APSE-like. However, this result should be taken with caution because the DNA polymerase found in this predicted phage region is highly similar to DNA polymerases encoded by members of the genera *Serratia*, *Yersinia*, *Erwinia* and others.

CRISPR (clustered regularly interspaced short palindromic repeats) arrays and their associated proteins form adaptive immune systems that are present in most archaea and many bacteria and act against invading genetic elements (e.g., viruses and plasmids) (Makarova et al., 2011). CRISPR arrays were detected only in the genomes of the culturable strains SsAf2.3, SsAf24.1 and SsApa8A1, and Cas protein sequences have been found only in the SsAf24.1 genome (**Table S8**). These results suggest that the transition from a free-living to a host-restricted lifestyle also led to the elimination of the CRISPR defense mechanisms.

### Secretion Systems Encoded by *S. symbiotica* Genomes

The three culturable *S. symbiotica* strains have pathogenic effects on infected aphids (Pons et al., 2019b; Elston et al., 2020; Perreau et al., 2021). The SsAf2.3 strain is also endocytosed into early embryos during ontogenesis and is compartmentalized into *A. pisum* sheath cells in a similar fashion as mutualistic strains, but ultimately fails to establish a stable association with aphids by vertical transmission (Perreau et al., 2021). This could be due to the still too high degree of pathogenicity of these strains, which hinders the establishment of stabilized associations. Secretion systems are among the many mechanisms used by pathogenic and symbiotic bacteria for host colonization and contribute to their degree of virulence: they allow bacteria to translocate effector proteins in host cells to modulate the host environment, thereby facilitating host invasion (Costa et al., 2015). In contrast to the facultative and co-obligate intracellular strains, the three culturable strains retain an intact type III secretion system (T3SS) (**Table S9**). These T3SSs are similar in sequence, gene content and gene orientation; and it is likely that it was acquired *via* horizontal gene transfer. Further, they appear to be related to the T3SS from *Yersinia enterocolitica* 8081 and thus belong to the SPI1 family generally associated to infection of animal hosts (**Figure S4**) (Abby and Rocha, 2012). The acquisition of the T3SS through horizontal gene transfer is also supported by the lower GC content of the T3SS gene cluster (44.9%) compared to the rest of the genomes (51.47-51.99%). Widely distributed among proteobacterial pathogens, T3SSs can be required during the establishment of symbiotic relationships between bacteria and eukaryotes (Göttfert et al., 2001; Dale et al., 2005), as in the case of tsetse fly invasion by the symbiont *Sodalis glossinidius* (Dale et al., 2001; Dale et al., 2002).

In addition, all *S. symbiotica* strains also exhibit several type V secretion systems (T5SSs). T5SSs are autotransporters that secrete virulence factors, often involved in cell-to-cell adhesion and biofilm formation (Leo et al., 2012). Their potential role(s) in the context of symbiotic relationships has however been poorly studied. Interestingly, SsApa8A1 is the only culturable strain that encodes for a complete flagellum: the genomes of SsAf2.3 and SsAf24.1 harbor many flagellar-coding genes, but do not have the full set of genes required for the formation of a complete flagellum (**Table S9** and **Figure 5**). Given that the pathways for chemotaxis and motility are among the first to be altered in the transition from a free-living to a host-dependent lifestyle (Hendry et al., 2014), this suggests that compared to strains SsAf2.3 and SsAf24.1, SsAp8A1 is less advanced in the process of host-adaption and is still competent for swimming motility, which could facilitate access to new hosts or habitats. SsApa8A1 is also the only strain to harbor several T4SSs: sub-type I (T4SS-typeI) and protein secretion T4SS (pT4SSt). In addition to their ability to translocate effector proteins, T4SSs have the unique ability to mediate DNA translocation in bacterial or eukaryotic target cells (Costa et al., 2015). For example, the endosymbiont *Wolbachia*, but also the intracellular pathogens *Coxiella burnetii* and *Legionella* spp. use T4SSs to deliver ankyrin repeat containing proteins (ANKs) into the host cytoplasm (Segal et al., 2005; Pan et al., 2008; Nikoh et al., 2018). Intracellular symbionts of plants of the genus *Rhizobium* also use T4SSs to invade root cortical cells during nodulation and thus acquire an intracellular lifestyle (Deakin and Broughton, 2009). Ongoing studies should determine the role of the different macromolecular systems conserved in the genomes of culturable strains of *S. symbiotica* during the infection process in insect hosts, but also in plants.

### The Array of Virulence Factors Encoded by the *S. symbiotica* Genomes

**Figure 6:**
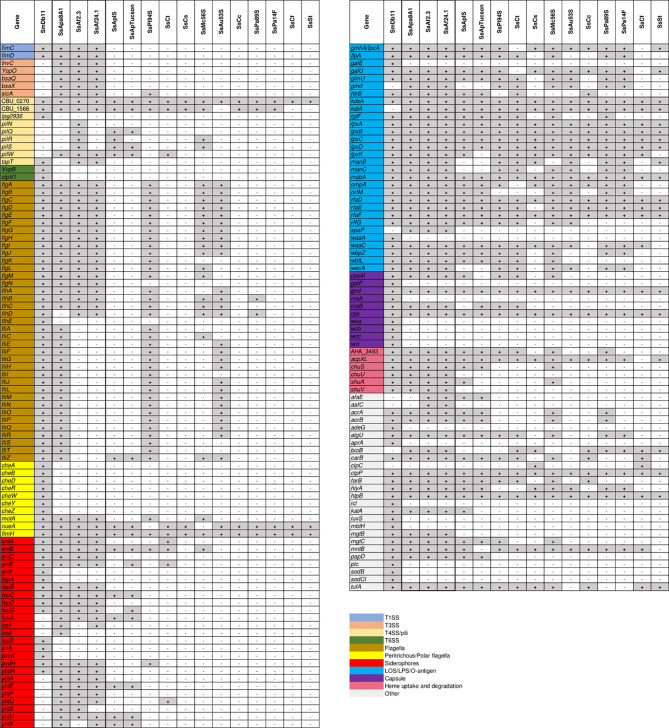
Presence/absence map of *S. symbiotica* virulence genes. Presence of a virulence gene is labeled in gray and absence in white.

The success of host colonization by invasive bacteria, whether in the host-pathogen relationship or in mutualistic symbiosis, is largely determined by the ability of microorganisms to interact with hosts through the expression of various colonization factors (Hentschel et al., 2000; Bright and Bulgheresi, 2010). Strains SsAf2.3, SsAf24.1 and SsApa8A1 are capable of rapidly invading the digestive tract of aphids and, as shown for SsAf2.3, the embryos when it is microinjected into the hemolymph, ultimately affecting insect survival and reproduction (Renoz et al., 2015; Pons et al., 2019b; Elston et al., 2020). These observations suggest that these strains are well equipped to invade and persist in host tissues. Compared to the entomopathogenic bacterium *S. marcescens* Db11 (SmDb11), the genomes of the culturable *S. symbiotica* strains harbor a much narrower range of virulence genes. However, compared to the host-restricted *S. symbiotica* strains, they retain a greater diversity of virulence factors including siderophore-related proteins and T3SS effector proteins (**Figure 6**; see **Table S10** for subject description & VFDB reference). This diversity in virulence factors may explain their pathogenicity and why they fail at this stage to establish a stable relationship by maternal transmission when infecting the aphid hemolymph (Perreau et al., 2021).

Interestingly, the three culturable *S. symbiotica* strains share most of the potential virulence genes, suggesting that they are adapted to similar environments. Their genomes have in common the presence of many genes involved in siderophore-mediated iron transport such as yersinabactin and enterobactin. Iron uptake by siderophores promotes host colonization by many pathogens, but also by symbiotic bacteria such as *S. glossinidius* during the invasion of the tsetse fly (Smith et al., 2013a). The genomes of the three culturable strains also harbor many genes involved in the expression of fimbrial adhesins that may be involved in host adhesion and biofilm formation (Gerlach and Hensel, 2007). All three genomes harbor *ilpA*, a gene coding for an adhesin used by many gut-pathogens for host invasion (Lee et al., 2010). Several genes coding for secreted effector proteins have been detected in all three genomes, including YopO (also known as YpkA), a T3SS effector protein used by pathogenic *Yersinia* species to escape the host immune system by inducing actin-filament disruption during phagocytosis (Lee et al., 2015). The genes *bsaQ* and *bsaX*, also found in all three genomes, code for T3SS effector proteins that have been found essential for intracellular survival of *Burkholderia pseudomallei* in host cells (Muangsombut et al., 2008). Few toxin production-related genes were detected, with the exception of genes encoding the hemolytic protein hemolysin III and the pore-forming toxin *Vibrio cholerae* cytolysin (VCC) encoded by *hlyA*, both associated with a cytolytic/cytotoxic activity against wide range of eukaryotic cells (Baida and Kuzmin, 1996; Kathuria and Chattopadhyay, 2018). Intriguingly, all strains seem to have retained some ability to synthesize T4SS effectors while, with the exception of SsApa8A1, they did not retain any complete T4SS. Finally, although SsApa8A1 appears to be the only one of the three culturable strains to have retained the ability to construct a complete flagellum, the genomes SsAf2.3 and SsAf24.1 harbor many flagella-related genes. Many symbiotic bacteria have preserved flagellar basal bodies (FBBs) that are functional type III secretion systems (Moya et al., 2008). However, the presence of such systems in SsAf2.3 and SsAf24.1 remains uncertain because many genes required for the acquistion of such systems appear to be absent (e.g. *fliF*, *fliG*, *fliH*, *fliN*, and many others).

Most of the virulence genes common to the fifteen *S. symbiotica* genomes are involved in the biosynthesis of lipopolysaccharides (LPS) and lipooligosaccharides (LOS), the main components of the outer membrane of gram-negative bacteria that can mediate microbe-host interactions (Bright and Bulgheresi, 2010; Poole et al., 2018). The gene *htpB*, known for encoding a multifunctional heat-shock protein involved in the internalization of certain pathogens in host cells (Garduno et al., 2011), is also conserved in all the *S. symbiotica* genomes as well as *clpP*, a gene coding for a serine protease required for intracellular parasitism of macrophages by *Listeria monocytogenes* (Gaillot et al., 2000). Very little is known about the virulence factors necessary for the intracellular lifestyle of insect mutualistic symbionts. These two genes undoubtedly represent excellent candidates to tackle the genetic basis for the internalization of bacterial symbionts in host cells. How virulence factors are modulated during host invasion and internalization in host cells remains enigmatic in the context of bacterial mutualism in insects, and culturable *S. symbiotica* strains at the pathogen-symbiote interface, which are easier to manipulate than host-limited strains, should greatly ease the study of these mechanisms.

## Conclusion and Perspective

Recently, several strains of *S. symbiotica* living in the guts of *Aphis* species have been isolated and cultivated. These strains probably evolved from plant-associates and could be, at this stage, involved in multi-host associations as has been reported for several phytopathogenic bacteria (Nadarasah and Stavrinides, 2011). Associated with pathogenic effects in aphids, these strains are remarkable because they may be a sibling group of the presumed ancestors of mutualistic *S. symbiotica* strains that have evolved towards a host-restricted lifestyle and thus provide a rare opportunity to decipher the genomic features of bacterial lineages before evolution towards host dependence. The three culturable strains show signs of early genome reduction but still have a gene pool that allows them to colonize new hosts, suggesting that they are intermediate stages between a free-living and a host-restricted lifestyle. With their extended metabolic capacities, they could be a source of metabolic innovations for the infected insects. A growing number of examples show that bacterial mutualisms have been established in insects through independent transitions to endosymbiosis (Clayton et al., 2012; Smith et al., 2013b; Husnik and McCutcheon, 2016; Boscaro et al., 2017). They support the hypothesis that recent events of acquisition/replacement of symbionts from a common lineage of “progenitors” from the environment may evolve into host-dependent relationships. Recent work conducted on plant-sucking stinkbugs experimentally demonstrated that the specialized obligate symbiotic bacteria associated with these insects can be replaced by less specialized free-living bacteria residing in the environment (Hosokawa et al., 2016a; Hosokawa et al., 2016b). Aphid-*S. symbiotica* associations offer remarkable examples of independent acquisitions of a symbiont species that have led to the emergence of mutualistic associations that may be facultative or co-obligate (Manzano-Marín and Latorre, 2016). In aphid species of the subfamilies Lachninae and Chaitophorinae, ancient co-obligate *S. symbiotica* strains have often been repeatedly replaced by more recently acquired strains (Manzano-Marín and Latorre, 2014; Manzano-Marín et al., 2016; Manzano-Marín et al., 2017; Monnin et al., 2020). These repeated replacements assume the existence of *S. symbiotica* strains available in the immediate environment of the host such its diet. Currently, little is known about how pathogenic bacteria evolve to integrate a stabilized mutualistic relationship with an insect host. The availability of culturable *S. symbiotica* strains at the pathogen-symbiont interface as well as their genomic sequence thus open new experimental perspectives to understand the drivers for the emergence of heritable mutualistic symbioses between bacteria and insects.

## Data Availability Statement

The datasets presented in this study can be found in online repositories. The names of the repository/repositories and accession number(s) can be found in the article/**Supplementary Material**.

## Author Contributions

FR, VF, and TH conceived and designed the research. FR, VF, JA, PB-P, BB, AG, and PM performed the research. FR, VF, JA, GL, and PM analyzed the data. FR wrote the paper. VF, JA, BB, JM, PM, J-LG, FC, and TH made manuscript revisions. All authors contributed to the article and approved the submitted version.

## Funding

This study was financially supported by Grant FRFC 6886819 from the Belgian Funds for Scientific Research (F.R.S.-FNRS) and FRIA grant no. 1.E074.14). This paper is publication BRC268 of the Biodiversity Research Centre (*Université catholique de Louvain*). The funders had no role in study design, data collection and analysis, decision to publish, or preparation of the manuscript.

## Conflict of Interest

The authors declare that the research was conducted in the absence of any commercial or financial relationships that could be construed as a potential conflict of interest.
