# Supplementary material for: At the Gate of Mutualism: Identification of Genomic Traits Predisposing to Insect-Bacterial Symbiosis in Pathogenic Strains of the Aphid Symbiont Serratia symbiotica
Source: Front Cell Infect Microbiol. 2021 Jun 29;11:660007. doi: 10.3389/fcimb.2021.660007 (PMC8275996; doi:10.3389/fcimb.2021.660007)

Figure S1. Plasmid profiles on Eckhardt gel with *B. thuringiensis* AND508 used as molecular plasmid marker size (M2) with three plasmids (128, 235 and 350 kb), 1: *S. symbiotica* strain CWBI-2.3^{T} (SsAf2.3) with two plasmids.

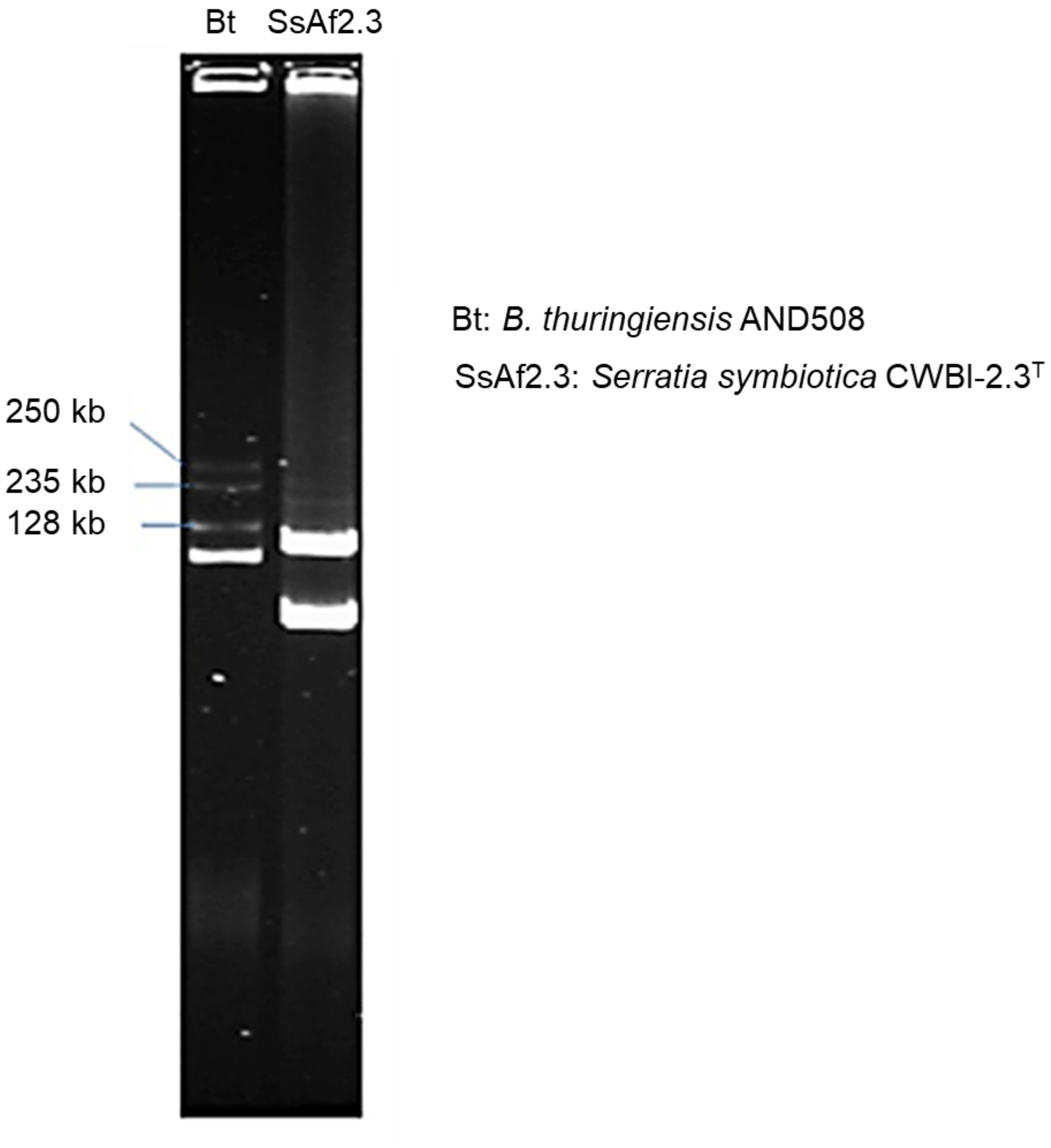

Supplement: Supplementary file 1 [file DataSheet_1.zip › Supplementary Material/Figure S1.pdf]
