# Supplementary material for: At the Gate of Mutualism: Identification of Genomic Traits Predisposing to Insect-Bacterial Symbiosis in Pathogenic Strains of the Aphid Symbiont Serratia symbiotica
Source: Front Cell Infect Microbiol. 2021 Jun 29;11:660007. doi: 10.3389/fcimb.2021.660007 (PMC8275996; doi:10.3389/fcimb.2021.660007)

Figure S2. Genome rearrangements undergone in culturable *S. symbiotica*. Pairwise synteny plot of free-living *S. marcescens* Db11, *S. ficaria* NCTC12148 along with culturable relatives *S. symbiotica* SsAf 2.3, SsAf 2.4 and SsAPA8A1. Red and blue lines connect blocks of more than 1kb in direct and inverted orientation. The stronger the line, the more nucleotide identity between synteny blocks.

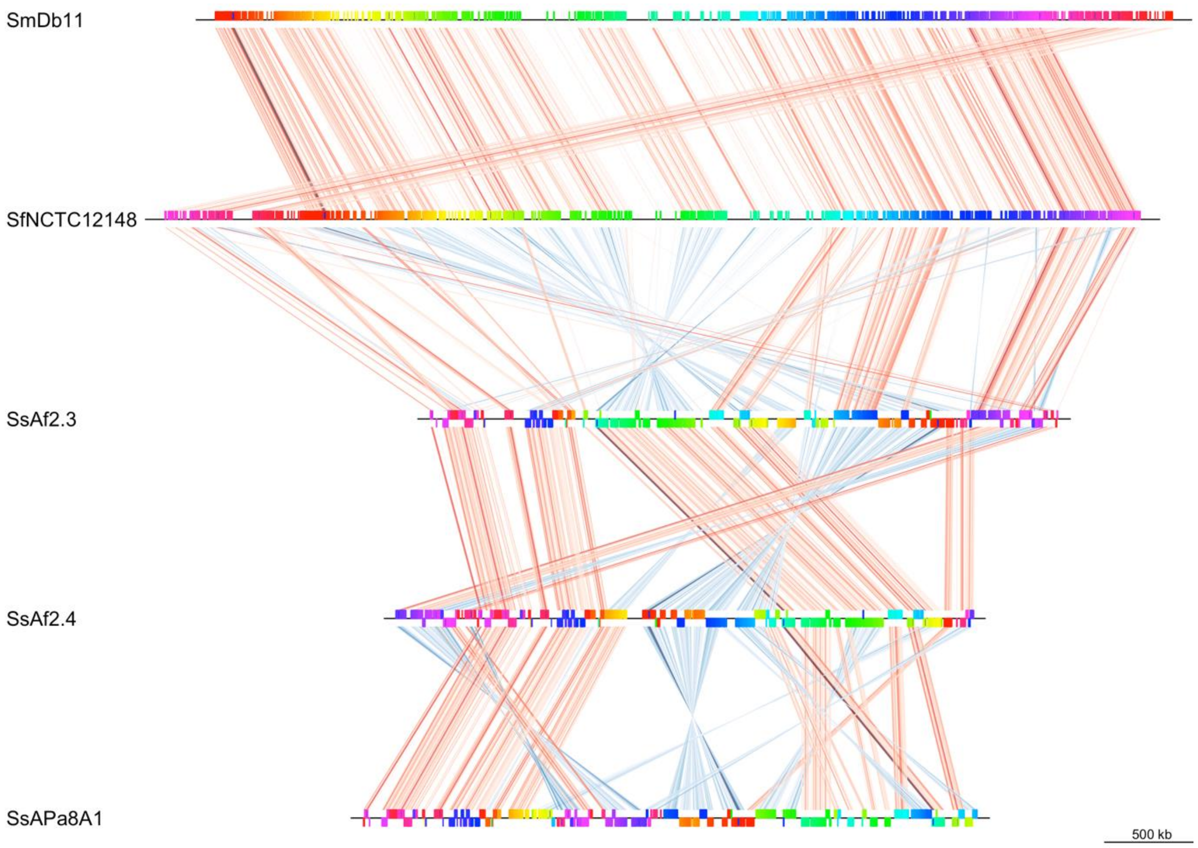

Supplement: Supplementary file 1 [file DataSheet_1.zip › Supplementary Material/Figure S2.pdf]
