# Supplementary material for: At the Gate of Mutualism: Identification of Genomic Traits Predisposing to Insect-Bacterial Symbiosis in Pathogenic Strains of the Aphid Symbiont Serratia symbiotica
Source: Front Cell Infect Microbiol. 2021 Jun 29;11:660007. doi: 10.3389/fcimb.2021.660007 (PMC8275996; doi:10.3389/fcimb.2021.660007)

Figure S3. Synteny plot showing the conservation of *cvpA* in all *S. symbiotica* genomes (the absence of the genomic fragment comprising *cvpA* in Ssc-56S is probably the result of a technical artifact during genome assembly).

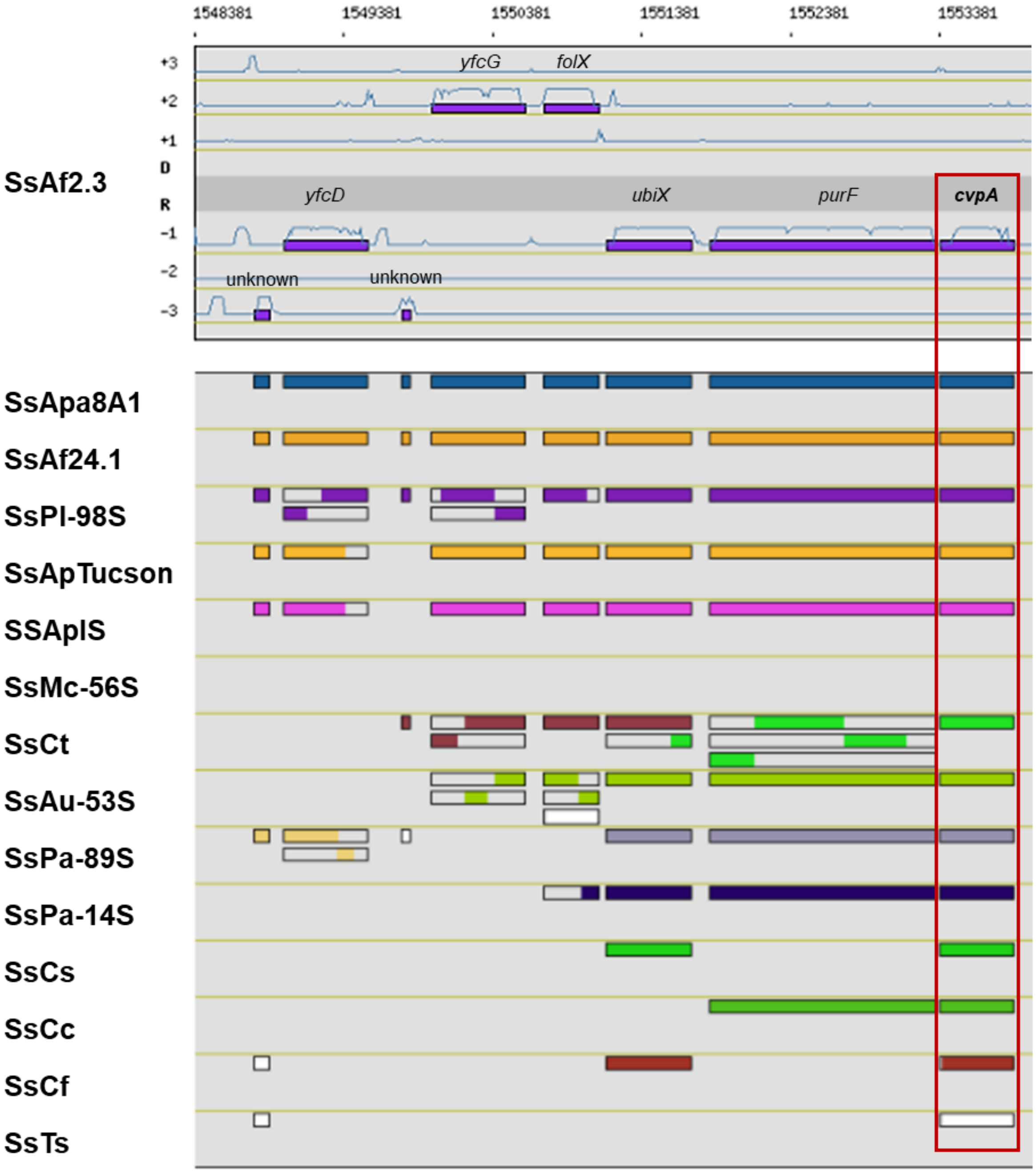

Supplement: Supplementary file 1 [file DataSheet_1.zip › Supplementary Material/Figure S3.pdf]
