# Supplementary material for: At the Gate of Mutualism: Identification of Genomic Traits Predisposing to Insect-Bacterial Symbiosis in Pathogenic Strains of the Aphid Symbiont Serratia symbiotica
Source: Front Cell Infect Microbiol. 2021 Jun 29;11:660007. doi: 10.3389/fcimb.2021.660007 (PMC8275996; doi:10.3389/fcimb.2021.660007)
Supplement: Supplementary file 1 [file DataSheet_1.zip › Supplementary Material/Figure S4.pdf]

Figure S4. Origin of the type III secretion system in culturable *S. symbiotica*. A. Phylogenetic relationship between T3SS of selected pathogens and culturable *S. symbiotica*. The tree shown is the maximum likelihood topology inferred on the concatenated protein sequences of SctC, SctT, SctU and SctV, composing the core of non-flagellar T3SS (Abby and Rocha, 2012). Boostrap values are given at tree nodes. B. Alignment of T3SS in *S. symbiotica* SsAf2.3 and *Y. enterocolitica* 8081. Lines connecting the sequences schematics indicate regions of similarity and percentages indicate the range of similarity in the different portions.

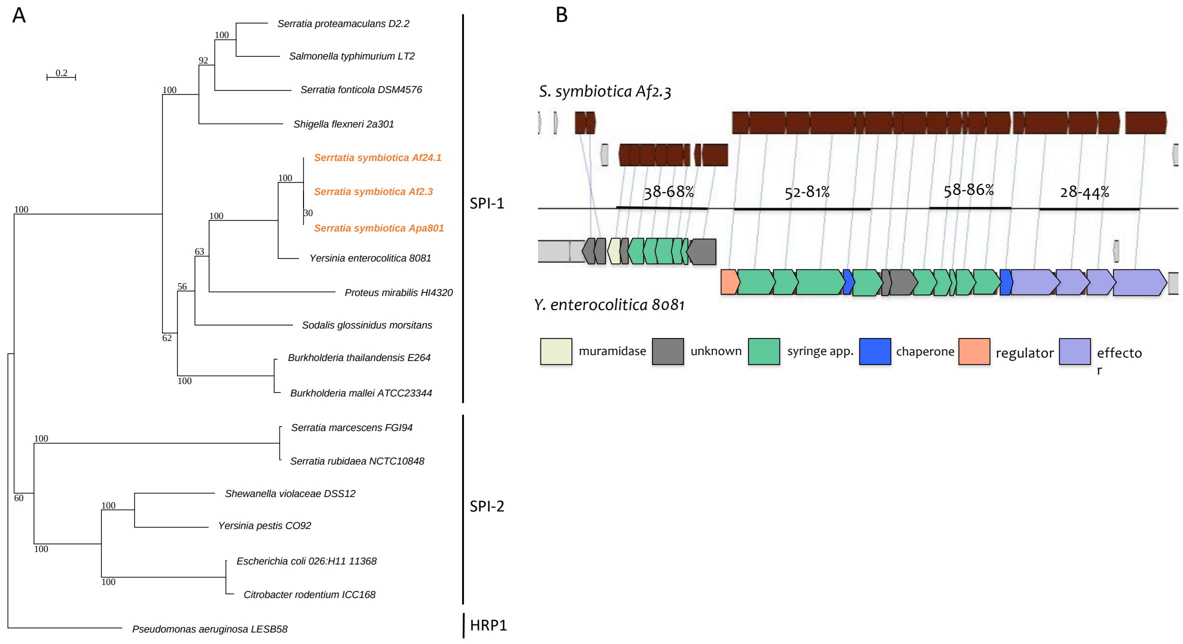

M&M supp
Phylogeny of T3SS

For all *Serratia sp.* carrying a T3SS and twelve representative bacterial genomes, we collected and concatenated protein sequences of SctC, SctT, SctU and SctV genes belonging to the NF-T3SS core proteins [1]. Then the concatenated sequences were aligned using the MUSCLE program in SeaView v5.0.4 [2]. LG+I+G model was selected as the best fit evolutionary model using PROTTEST v3.4.2 [3] and the phylogenetic tree was built using the maximum likelihood method with PHYML v3.1 [4]. Nodal support was evaluated with 100 bootstrap replications.

References

1. Abby, S.; Rocha, E.P.C. The non-flagellar type III secretion system evolved from the bacterial flagellum and diversified into host-cell adapted systems. *PLoS Genet.* **2012**, *8*, e1002983, doi:10.1371/journal.pgen.1002983.
2. Gouy, M.; Tannier, E.; Comte, N.; Parsons, D.P. Seaview Version 5: A Multiplatform Software for Multiple Sequence Alignment, Molecular Phylogenetic Analyses, and Tree Reconciliation. In *Methods in Molecular Biology*; 2021; pp. 241–260.
3. Darriba, D.; Taboada, G.L.; Doallo, R.; Posada, D. ProtTest 3: Fast selection of best-fit models of protein evolution. *Bioinformatics* **2011**, *27*, 1164–1165, doi:10.1093/bioinformatics/btr088.
4. Guindon, S.; Dufayard, J.F.; Lefort, V.; Anisimova, M.; Hordijk, W.; Gascuel, O. New algorithms and methods to estimate maximum-likelihood phylogenies: Assessing the performance of PhyML 3.0. *Syst. Biol.* **2010**, *59*, 307–321, doi:10.1093/sysbio/syq010.
